# Roux-en-Y Gastric Bypass Surgery Leading to Postprandial Hypoglycemia: A Case Report

**DOI:** 10.7759/cureus.32265

**Published:** 2022-12-06

**Authors:** Telmo Coelho, Andreia Freitas, Henrique Carmona Alexandrino, Sara Pinto

**Affiliations:** 1 Internal Medicine, Centro Hospitalar Vila Nova de Gaia | Espinho, Vila Nova de Gaia, PRT; 2 Endocrinology, Diabetes and Metabolism, Centro Hospitalar Vila Nova de Gaia | Espinho, Vila Nova de Gaia, PRT

**Keywords:** postprandial hypoglycemia, micronutrient deficiency, iron deficiency, roux-en-y gastric bypass, prothrombin related thrombophilia

## Abstract

Postprandial hypoglycemia is a rare complication after Roux-en-Y gastric bypass (RYGB). The underlying pathophysiology remains to be fully understood.

We present a case of a 49-year-old woman with a past medical history of mesenteric thrombosis due to prothrombin-related thrombophilia, which culminated in RYGB 10 years prior to presentation. The patient had been given anticoagulation treatment for several years, which she abandoned one year prior to presentation. She presented to our consultation with episodes of postprandial hypoglycemia and severe anemia due to iron and vitamin B12 deficiencies.

Dietary adjustments were set in place to prevent hypoglycemia and neuroglycopenic symptoms. Intravenous iron and intramuscular vitamin B12 supplementation led to full recovery of hemoglobin levels, allowing restart of oral anticoagulation to prevent recurrence of thrombotic events.

## Introduction

Prothrombin-related thrombophilia is the second most common genetic form of thrombophilia, just after factor V Leiden thrombophilia [1,2]. Some studies estimate its prevalence in the U.S. and European population to be around 1.7-3% [1,2]. Main manifestation of prothrombin thrombophilia is deep vein thrombosis and pulmonary embolism [2]. Nevertheless, other vascular beds can be affected, such as mesenteric veins or arteries [3], which can require surgical treatment. Prothrombin-related thrombophilia requires anticoagulation. The duration of anticoagulation therapy depends on the number of thrombotic events, risk for recurrence, and anticoagulant-related bleeding risk [1,2].

A life-threatening complication of hyperinsulinemic hypoglycemia has been reported in a minority of patients that underwent Roux-en-Y gastric bypass surgery (RYGB) [4-8]. The underlying physiopathology remains not fully understood, but RYGB leads to earlier and higher glucose peaks, which are responsible for large insulin and glucagon-like peptide 1 (GLP-1) secretion that causes a lower glucose nadir [4-8]. This complication usually presents with neuroglycopenic symptoms several years after surgery [4-8]. Available therapeutic options are extremely limited; therefore, dietary modifications, such as avoiding simple carbohydrates, and adding protein and fat to every meal to reduce the glucose excursion and hypoglycemia, remain the cornerstone of treatment [4,5].

The importance of the duodenum, initial jejunum, and gastric intrinsic factor in iron, folate, vitamin B12, and other nutrients absorption is well documented. RYGB can disturb the normal digestive process and induce nutritional deficiencies and anemia [9-12]. A meta-analysis conducted by Weng et al. linked RYGB to an increased risk of anemia and deficiencies of iron and vitamin B12 [9]. In recent years, several studies [10-12] furthermore elucidated the consequences of RYGB in iron and micronutrients absorption. Thus, regular follow-up with comprehensive nutrient profiles of all RYGB patients is of utmost importance.

## Case presentation

We present the case of a 49-year-old woman, who was referred to our Internal Medicine consultation due to anemia of unknown etiology and recurrent thrombotic events. She had a past medical history of prothrombin-related thrombophilia (20210G>A heterozygosity) with recurrent deep vein thrombosis of both femoral veins and one episode of mesenteric thrombosis, which complicated and culminated in an RYGB 10 years prior to presentation. She had been on prolongated anticoagulation with rivaroxaban during several years, nevertheless she abandoned treatment one year prior to our first medical appointment.

She was referred to our consultation for hypochromic and microcytic anemia with a hemoglobin level of 6.1g/dL, which was fortuitously discovered in a pre-surgical work-up for a fractured radius after a work accident.

At our consultation, the patient complained of asthenia and dizziness for several weeks. She had regular catamenia and no known history of blood loss. Her diet was rich in fruits and vegetables without any food restriction or intolerance. She had already been taking oral iron supplementation, which did not improve iron or hemoglobin levels.

Besides these complaints, the patient revealed that she had several episodes of symptomatic hypoglycemia (with reported glucose levels between 35mg/dL and 60mg/dL) during the past 8 years, despite not being diabetic or on any medication. She had been monitoring her plasma glucose levels and referred to neuroglycopenic symptoms appearing a couple of hours after a meal rich in carbohydrates.

On physical examination, her blood pressure was 122/69mmHg, heart rate was 77 beats per minute, and oxygen saturation level of 98% at room air. She had slightly discolored skin and mucous membranes. Cardiac and pulmonary auscultations were normal.

We performed a blood test, which showed a microcytic, hypochromic anemia (Hb of 5.4mg/dL, mean globular corpuscular of 63.0fL, mean corpuscular hemoglobin concentration of 27.1pg/cell) with severe iron and vitamin B12 deficiency (total iron of 10µg/dL, ferritin of 3.5ng/mL, transferrin saturation of 2.2%, total iron-binding capacity of 451µg/dL, vitamin B12 of 161.0pg/mL). Vitamin D levels were also low (vitamin D 25nmol/L). Haptoglobin, lactate dehydrogenase, and folate levels were normal. Renal function, hepatic enzymes, thyroid function, and protein electrophoresis were normal. Glycated hemoglobin was 4.4%. Peptide C dosing was also normal.

The patient underwent continuous glucose monitoring after high carbohydrate ingestion. At 2 hours after ingestion, she reported neuroglycopenic symptoms, which corresponded to blood glucose levels of 31mg/dL.

A cervicothoracoabdominopelvic CT scan and gynecologic ultrasound found no significant alterations. High digestive endoscopy and colonoscopy were also normal.

Anemia due to iron and vitamin B12 was supplemented with 1000mg + 1000mg of intravenous iron and monthly intramuscular vitamin B12 administration. Oral vitamin D supplementation were started as well. An analytic control a few months later showed complete recovery of anemia with normal iron and vitamin B12 levels. This allowed the reintroduction of oral anticoagulation to prevent thrombotic events. The patient is now on maintenance therapy with monthly intramuscular vitamin B12 and regular intravenous iron supplementation, which is guided by ferritin levels. To prevent postprandial hypoglycemia, our patient started dietary modifications and was referred for nutrition and endocrinology consultation.

## Discussion

Prothrombin-related thrombophilia can cause recurrent thrombotic events, mostly deep vein thrombosis and pulmonary embolism [1,2]. 20210G>A heterozygosity alone is not an indication for long-term anticoagulation [2]. Further studies are mandatory to assess the safety and efficacy of direct oral anticoagulants versus warfarin in these patients.

Despite not being the most common manifestation, other vascular beds can be affected, such as mesenteric veins or arteries [3]. Our patient had a complicated mesenteric thrombosis 10 years prior to presentation, which terminated in an RYGB. This thrombotic event was the start to a complex physiopathological process resulting in an impaired digestive process and several nutrient deficiencies.

In consultation, our patient mentioned postprandial episodes of dizziness and confusion, starting a few years after RYGB. She later found out that these episodes were linked to hypoglycemia. Even though rarely reported, postprandial hyperinsulinemic hypoglycemia following RYGB is believed to be underdiagnosed [4,5]. This fact supports the need to maintain close vigilance on neuroglycopenic symptoms after RYGB. Dietary modifications remain the cornerstone of treatment [4,5].

In addition to postprandial hypoglycemia, RYGB is known to cause modifications in the normal digestive process and therefore induce nutritional deficiencies and anemia [9-12]. Our patient presented with severe anemia due to iron and vitamin B12 deficiencies. Disruption of gastric end enteric absorption of these nutrients implies that supplementation must be done intravenously and intramuscularly.

Regardless of our patient’s prothrombin-related thrombophilia, we decided to postpone the restart of oral anticoagulation until colonoscopy and high digestive endoscopy were performed. Given the exclusion of occult hemorrhagic lesions and our patient’s multiple recurrences of thrombotic events, we decided to restart oral anticoagulation with rivaroxaban for undefined duration.

## Conclusions

With this case report, we highlight the importance of a detailed medical history and the capacity to integrate different problems in a holistic approach. Postprandial symptomatic hypoglycemia appearing after RYGB is believed to be underdiagnosed; therefore, a high clinical suspicion and active vigilance is required. Dietary modifications remain the best treatment to avoid neuroglycopenic symptoms and to reduce the risk of hypoglycemia. Furthermore, RYGB can cause anemia due to iron and vitamin B12 deficiencies requiring parenteral supplementation. Our patient had also a prothrombin-related thrombophilia requiring restart of anticoagulation after exclusion of occult hemorrhagic lesions. Further studies are needed to evaluate safety and efficacy of direct oral anticoagulants versus warfarin in these patients.
